# Assessment of internal fit and micro leakage of conventionally fabricated ceramometallic restoration versus CAD wax and press veneering (in-vitro study)

**DOI:** 10.1038/s41405-021-00072-7

**Published:** 2021-05-10

**Authors:** Fatema Khaled Mansour, Rabab Mohammed Ibrahim, Hoda Mansour, Ahmed Mohamed Hamdy

**Affiliations:** 1 Faculty of Oral and Dental Medicine, October University of Modern Sciences and Arts, Cairo, Egypt; 2 Faculty of Dentistry, Cairo University, Cairo, Egypt; 3 University of Business and Technology, Jeddah, Saudi Arabia; 4 Faculty of Oral and Dental Medicine, October University for Modern Sciences and Arts, Cairo, Egypt

**Keywords:** Fixed prosthodontics, Non-bonded restorations

## Abstract

**Statement of problem:**

Accuracy of internal fit and microleakage for CAD-CAM systems used in metal coping fabrication and veneered with layering or pressing porcelain in ceramometallic restoration is unclear.

**Material and methods:**

A master metal die was milled to resemble the right mandibular first molar preparation for coverage with ceramometallic restoration. Master die was duplicated to twenty-four resin specimen dies.They were divided into two groups according to metal coping construction technique using either conventional (C) or CAD (D) wax. Each group was subdivided into two subgroups (*n* = 6) according to the technique of porcelain veneering (layered or pressed) to fabricate ceramometallic restorations, where subgroup (CL, DL) were conventionally layered by porcelain and (CP, DP) were press veneered. A standardized thickness of metal and porcelain was performed in all specimens as per manufacturer’s instructions for techniques ceramometallic restoration construction.

Evaluation of internal fit was done with silicone replica technique using stereomicroscope at ×24 magnification where the thickness of silicon layer was measured at 20 reference points on each specimen. Then specimens were subjected to thermocycling. Sectioned specimens were assessed for microleakage using a stereomicroscope at ×12 magnification along die-cement interface with a five scale score.

**Results:**

Mean internal gap values of veneering showed a statistically nonsignificant difference between specimens made with layering(L) and pressing(P). Different techniques of wax construction showed a non-significant difference in internal gap values between specimens made with conventional(C) and CAD(D) waxing. However, a significant difference was found in the internal gap at different sites. The highest internal gap was found at the occlusal surface, while the lowest gap was found at the finish line. The highest mean microleakage score was found with CAD wax and press veneering, while the lowest mean microleakage score was found with conventional wax and press veneering.

**Conclusion:**

Both construction techniques of ceramometallic restoration were considered reliable in restoration production within a clinically acceptable range regarding internal fit and microleakage. There is a strong positive correlation between internal fit and microleakage of ceramometallic restoration constructed.

## Introduction

Fit of a restoration is determined by its marginal and internal fit. Marginal gaps result in gingival irritation, cement dissolution and recurrent decay.^1^ Internal fit is defined as the perpendicular distance between the framework and abutment teeth and the misfit is measured from occlusal/incisal and axial surfaces to the coping. It is a direct measure of the cement film thickness under restoration, and it is influenced significantly by the accuracy of the fabrication process.^2^ Internal fit ranging between 50 and 100 µm is considered acceptable.^3^ Despite new innovations in restorative materials and techniques to enhance clinical success, microleakage persists as one of the main biological causes for restoration failure.^4^ Microleakage is defined as the penetration of substances, such as oral fluids, bacteria into a structural defect that occurs between the restoration and tooth structure.^5^ Direct microscopic examination was the most commonly used method, followed by the silicon replica technique observed under the microscope. Besides, cross-sectioning of cemented specimens could be done. However, it provides a limited number of sections for a specimen.^6^ Microleakage tests provide evidence about the performance of restoration.^4^ Different techniques were developed to assess microleakage, including use of dyes, thermocycling, radioactive isotopes, air pressure, bacteria, neutron activation analysis, and artificial caries. For quantitative assessment of microleakage, dye penetration is measured using different microscope types.^4,5^

Insufficient scientific evidence was reached on the correlation between the accuracy of internal fit and microleakage for different fabrication techniques of metal coping using conventional and CAD wax followed by veneering with conventional layering and pressing porcelain on metal for construction of ceramometallic restoration.^7^

The conventional technique for fabricating metal substructure is the lost-wax technique. The conventional process comprises multiple materials as well as clinical and laboratory stages, leading to unavoidable inaccuracies.^2,8,9^ Wax–up quality is dependent on the skill of the operator. CAD/CAM enhanced the accuracy of standardized restorations and reduced errors, time, labor and production cost. However, CAD systems also have some disadvantages. Limitation of finite resolution in light laser scanner can make sharp edges slightly rounded.^1,2^

Layering technique is the principal method for veneering metalcore. Porcelain powder is mixed with modeling liquid, and the applied layer is overbuilt with a brush to compensate for firing shrinkage. This requires skill, multiple applications, and firings.^10^ Press to metal porcelain offers a solution for most of the disadvantages with PFMs.^11^ Firing shrinkage is minimized, resulting in a better fit with decreased porosity of porcelain by the support of the investment.^11^

This study aimed to compare the internal fit and the microleakage of ceramometallic restoration constructed using conventional waxing up and veneer porcelain layering technique versus CAD wax and porcelain press veneering.

The null hypothesis assumes that there will be no differences in internal fit and microleakage scores of restorations constructed with different techniques. The null hypothesis suggested that there will be no difference in different measuring sites of internal fit.

## Materials and methods

A master metal die was milled (with dimensions of 5.5 mm in height, 9.5 mm cervical diameter, 8 mm occlusal diameter, 1.2 rounded shoulder finish line with an axial taper of 7° and two planes were made occlusally) to resemble preparation of mandibular first molar for construction of full coverage ceramometallic restoration.^12,13^ Metal die was duplicated to 24 resin dies (Kemapoxy resin, CMB, Egypt) using polyvinylsiloxane duplication material (Ecosil, Dentarum, Germany)^14^ and randomly divided into two equal groups according to method of metal coping construction using conventional (C) or CAD (D) wax.^15–17^ According to previous studies^15,16^ 12 specimens were calculated in each group representing an 80% power. The study’s power was calculated using power analysis software (G*Power) ^18^ to calculate the number of specimens. Each group was subdivided into two subgroups with six specimens in each subgroup according to the porcelain application technique; (CL and DL) represented conventionally layered porcelain specimens while (CP and DP) were press veneered.

**Fig. 1:**
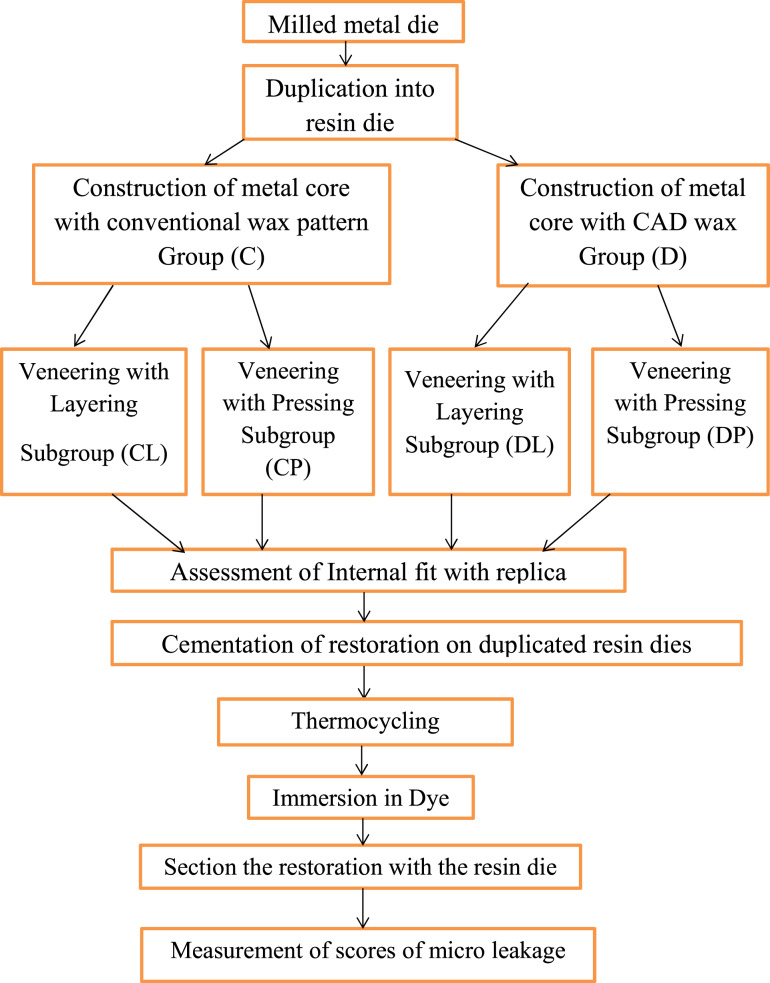
Study design. Schematic diagram illustrating briefly in-vitro study.

Resin dies were randomly allocated equally to subgroups utilizing (www.random.org). Blinding was not feasible for the operator because different techniques of restoration construction were used. However, the assessor and the statistician were blinded during the measurement of both internal fit and microleakage. The study design was illustrated in Fig. 1.

For the construction of ceramometallic restoration, two types of wax were used. In conventional wax (C), four coats of die spacer (Picofit, Renfert, Germany) (each coat was 13 µm × 4 = 52 µm) were applied on resin die followed by dipping of die in wax dipping pot (JT1402, Denshine, China). Then sculpting opaque gray wax (GEO Crowax, Renfert, Germany) was added to thin areas by electric wax knife (SJK 110, Bonew, USA) and margination with cervical red wax (GEO Crowax, Renfert, Germany). Verification with a wax calliper (Meta Dental IC# - 14401, Unique Dental Supply, Canada) to have 0.4 mm wax coping thickness. For CAD wax (D) (Yamahachi, Gamagori, Japan), the die was scanned by the dental scanner (Identica T-300, Medit DT, Korea) after antireflection spraying (Okklu-exact, Shera, Germany). Then, images were transferred to create a 3D virtual die. Exocad CAD software program (Exocad version 6136, Fraunhofer Institute, Germany) was used to digitally design coping with 0.4 mm thickness and 52 µm die spacer away 1 mm from the margin. CAD wax was milled by a dry milling machine (K5, Vhf, Germany). NiCr alloy (remanium, Dentaurum, Germany) was used for casting (Fornax T, Bego, Germany) of a metal substructure.

Layering and pressing ceramic veneering materials were used. For standardized buildup thickness of porcelain, first molar restoration was completed to full anatomy with dimensions of 1.2 mm cervically and 1.5 mm occlusally. Silicon index impression mold (Ecosil, Dentaurum, Germany) was made to standardize veneering porcelain for subgroups. Two layers of opaque paste form were applied for layered and pressed porcelain subgroups to have 0.1 mm thickness. For CL and DL subgroups, freehand build-up of porcelain (IPS InLine conventional layering, Ivoclar Vivadent,Germany); the first layer was made from dentin, and enamel powder which were mixed with build-up liquid then built on the opaque layer with brushes (Tanaka, USA), condensed and checked by previous mold index before firing. The thickness of 0.7 mm cervically and 1.0 mm occlusally was verified with a caliper (Thickness gauge dial, Unique dental supply, Canada).

For CP and DP subgroups, sculpting wax was used to build up restoration anatomically and checked by previous mold index as well before pressing of ingots (IPS InLine PoM, Ivoclar Vivadent, Germany). Recommended manufacture’s instructions for thickness of 0.7 mm cervically and 1.0 mm occlusally was built to compensate for porcelain shrinkage and provide better esthetics. Wax-ups were sprued, invested (IPS PressVEST Premium, Ivoclar Vivadent, Germany) and burnout (Vulcaan TM 3–130, Neytech, Canada) followed by pressing in the ceramic furnace(Programat EP3010, Ivoclar Vivadent, Germany). After cooling, fine divestment using Al_2_O_3_ with 50 μm glass polishing beads machine (Lianmei, Shenzhen Qianmei, China) with 1.5 bar pressure at a distance of 10 cm to be careful with shoulder margin area.

**Fig. 2:**
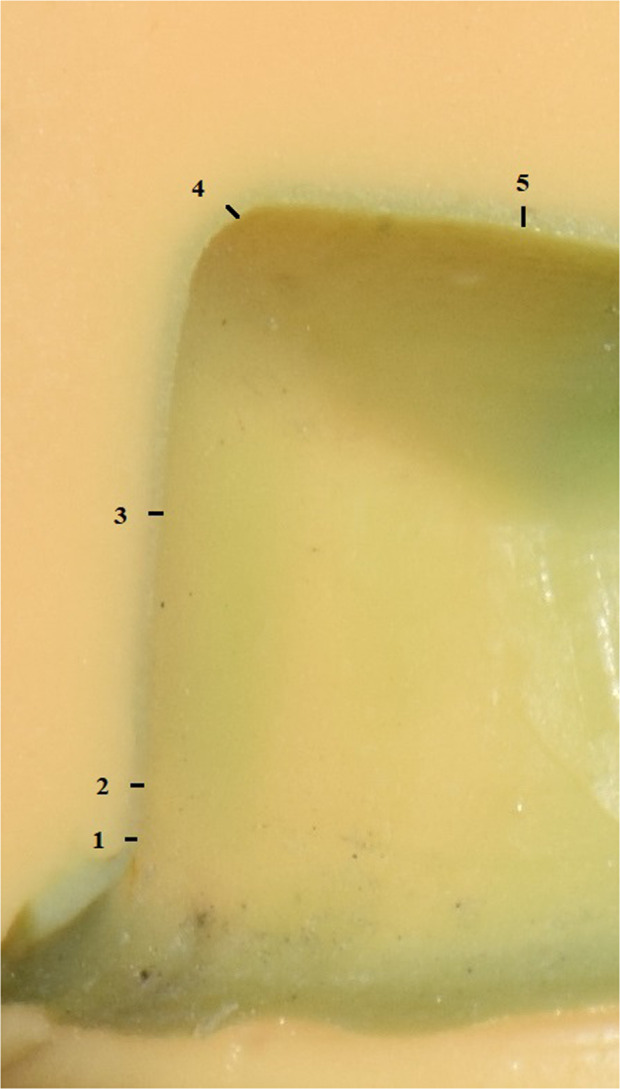
Internal fit specimen. Silicon replica specimen with five reference points for surface quadrant.

The internal fit was measured using silicon replica where light body (Elite HD, Zhermack, Italy) was applied in fitting the surface of restoration.^2,8,19^ Then putty body (Elite HD, Zhermack, Italy) was used to hold replica from the resin die surface. Each quadrant had 5 measured reference points using stereomicroscope (Technival 2, Carl Zeiss, Germany) at ×24 magnification Fig. 2. Images were taken using digital camera (Nikon D5300, Thailand) and analyzed using Image J analysis (Image J1.47, National Institute of Health, USA). An overall of 20 reference points were measured in μm for each specimen for four surfaces (Buccal, Lingual, Mesial and Distal).

**Fig. 3:**
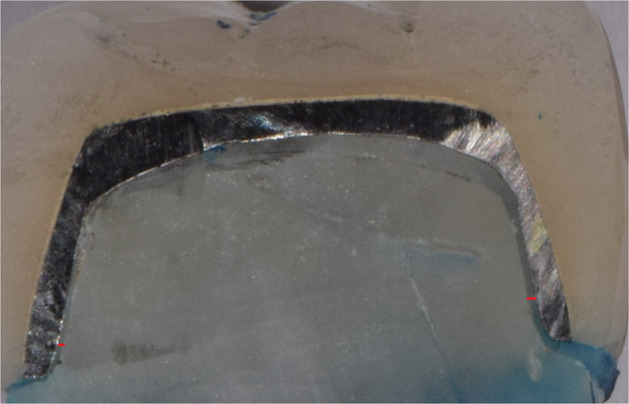
Microleakage specimen. Sectioned specimens showing microleakage scores of (1) and (2).

All ceramometallic restorations specimens were cemented with resin cement (G-Cem TM Capsules, GC, USA) onto their respective resin dies using standard loading device.^8^ Varnish was applied on all surfaces of specimens except for the finish line.^20^ Then the complex restorations-dies were thermocycled (SD Mechatronic thermocycler, Whaledent, USA) (5–55 °C) for 5000 thermal cycles which resembles 6 months service in the oral cavity.^21^ Specimens were then immersed in 2% methylene blue dye for 24 h, sectioned mesiodistally centrally by diamond saw (Isomet 4000, Buehler, USA)^5,22^ and viewed under stereomicroscope with ×12 magnification where dye penetration was measured along with die-cement interface and scored for microleakage using 5 score scale by Tjan et al.^5,20,23^ (Fig. 3).

## Results

Results were evaluated for normality by checking data distribution. Parametric data of internal gap width were analyzed using three-way ANOVA followed by comparison of main groups utilizing Bonferroni correction. The microleakage scores were analyzed using Mann–Whitney *U* test. The spearman rank order correlation coefficient was used to study the correlation between the internal gap and microleakage scores. The significance level was set at *P* ≤ 0.05.

The highest mean internal gap was apparent with CAD wax and press porcelain at the occlusal surface, while the lowest mean internal gap was found with conventional wax and layering porcelain (Table 1).

**Table 1:** Descriptive statistics for internal gap width (µm).

Position	Waxing technique	Veneering technique	Mean	Std. deviation
FL	Conventional	Layering	53.41	1.79
		Pressing	53.85	0.72
	CAD	Layering	53.73	1.70
		Pressing	53.65	1.06
Above FL	Conventional	Layering	70.81	2.72
		Pressing	71.27	1.51
	CAD	Layering	71.99	3.01
		Pressing	71.48	1.77
Axial	Conventional	Layering	99.68	5.26
		Pressing	102.33	6.91
	CAD	Layering	101.50	6.04
		Pressing	104.64	6.94
Axio-occlusal	Conventional	Layering	127.62	4.30
		Pressing	130.18	4.74
	CAD	Layering	125.46	2.00
		Pressing	130.06	5.99
Occlusal	Conventional	Layering	164.47	3.14
		Pressing	165.27	2.57
	CAD	Layering	163.58	4.67
		Pressing	164.95	3.55

Effect of the different variables: veneering, waxing, and measurement sites on internal gap width (μm) were presented in Table 2. Only the site of measurement had a significant effect on internal gap width (*p* < 0.001), while the effect of other variables was nonsignificant (*p* > 0.05).

All specimens showed degrees of microleakage scores. The highest mean microleakage score was found with CAD wax and porcelain press veneering (1.67), while the lowest mean microleakage score was found with conventional wax and press veneering (1.42) (Table 3).

There was a strong positive correlation between internal gap width and microleakage scores that was statistically significant (*r*_*s*_ = 0.764, *p* < 0.001).

## Discussion

The internal gap assessment has a clinical relevance affecting the strength of the restoration cement system.^2^ The occlusal fit significantly affects restorations’ structural durability, and the axial wall internal fit influences restoration retention.^1^ The internal fit measured by silicon replica is considered a reliable and non-destructive technique in in-vitro studies.^3,24^

The null hypothesis suggests no significant differences in internal fit and mircoleakage score values were accepted for different construction techniques. However, regarding the different measuring sites of internal fit, the other part of the null hypothesis was rejected due to a statistically significant difference in internal fit values.

The resin dies were used to simulate tooth structure as they have a similar modulus of elasticity to that of dentin (12.9 GPa)^14^ for proper wax wettability to epoxy dies which allow better wax pattern adaptation.^25^ The factors affecting internal fit include abutment preparation design, scanning device accuracy, software design, and milling machine accuracy in addition to wax fabrication techniques.^1,8^ Wax caliper verified thickness of wax for standardization.^15^ Porcelain veneer pressing on metal coping was suggested to provide better marginal adaptation^26^ as pressing removes voids and enhance crown accuracy.^27^

There was no significant difference in internal gap values between wax specimens because inaccuracies were equally weighed in both groups. This was found to be in accordance with some studies.^2,8,12^ CAD wax quality depends on software quality, milling precision of the virtual restoration and scanner that might elevate die geometry by rounding edges during scanning.^12^ Inaccuries related to conventional waxing up are related to operator skills and amount of die spacer placed.^8^

The results were in contradiction with Farjood et al.,^1^ where CAD wax experienced greater discrepancy due to the use of four-axis milling machine. Also, scanner might induce wider internal gaps due to use of antireflection spray.^28^

**Table 2:** Effect of the different variables on internal gap width (µm).

Variables	Sum of Squares	df	Mean Square	*F*	*p* value
Site	188684.5	4	47171.14	2935.6	<0.001*
Waxing technique	1.41	1	1.41	0.09	0.768 ns
Veneering technique	71.611	1	71.611	0.044	0.834 ns

There was no statistically significant difference in internal gap values between porcelain veneering specimens. This was in agreement with some studies^26,29^ that found that neither fabrication protocol nor repeated ceramic firings had any statistically significant effect on internal discrepancy values. The difference in veneering porcelain composition may have different coefficients of thermal expansion leading to inaccuracies.^30^ However, the use of the same ceramic system and the standardization of veneering ceramic thickness using silicon mold might have resolved inaccuracies equally.

The results were in contradiction with other studies that reported increased discrepancies due to structural changes in porcelain during firing at high temperature.^27,31^ *Fahmy and Salah*^27^ mentioned that although no statistically significant difference was evident, pressed crowns were not corrected during fabrication, while the crowns with the shoulder porcelain were corrected with a second firing.

Moreover, results showed there was a significant difference between internal gap values measured at different positions. The highest internal gap was found at occlusal surface followed by axio-occlusal area, while the lowest gap value was found at the finish line. The occlusal site might have the highest inaccuracy due to anatomical morphology, which may not allow the flow of replica with the accumulation of die spacer in the slope of prepared cusps.^8^ Also, high internal gap values at axio-occlusal site might be due to the milling machine’s inability to compensate for drill.^8,12^ The precision of internal fit depended on the size of the smallest tool used.^2^ While internal gap value was lowest at the finish line, convergence in preparation allows replica to flow easily and less reduction so errors are reduced.^15,28^ Results were in agreement with some studies^8,15,28^ and in contradiction with a study^32^ that found metal alloy casting uniformly shrinks at all measured regions as metal shrinkage and the investment expansion processes could influence the adaptation resulting in a homogeneous gap.

As for the internal gap measurement sites of both porcelain techniques, a significant difference was present. The highest discrepancy occurred at the occlusal area due to the maximum material thickness of restoration.

The results were in agreement with some studies,^26,33^ where the finish line and axial areas were subjected to more fitting during heat treatment and porcelain application. While in contradiction with a Kocaagaoglu et al.,^34^ that decreased occlusal gap after ceramic firing might be due to dimensional changes that may distort the underlying metal substructure.

The cementation using a loading device is preferred because the finger pressure might show variation.^5^ Resin cement was used to reduce microleakage.^20,22^ Thermocycling is a widely accepted method used in in-vitro microleakage studies.^4,21^ Placement in methylene blue dye provides high precision as dye has no fillers. Testing the leakage occurring at the die-cement interface was observed as it has greater biological significance.^4,20,35^

**Table 3:** Descriptive statistics for mean microleakage scores.

Waxing technique	Veneering technique	Mean	Std. deviation
C	L	1.50	0.52
	P	1.42	0.51
D	L	1.58	0.51
	P	1.67	0.49

There was no significant difference found in the microleakage score values between different construction techniques due to standardization. Results were in accordance with some studies^20,35^ that found resin cement had the lowest degree of microleakage. However, it was in contradiction with Kumar et al.,^5^ that high microleakage scores might occur with resin cement due to polymerization shrinkage combined with the coefficient of thermal expansion of materials during ageing.

The results showed a strong positive correlation between internal fit and microleakage for all subgroups and in agreement with studies.^23,36^ Sundar et al.,^23^ found that an increase in the marginal discrepancy will increase the microleakage proportionally, thereby increasing a fixed dental prosthesis’s failure rate. In contradiction Hooshand et al.,^37^ found no strong correlation between marginal fit and microleakage because of the significant variation of leakage scores due to multiple variables adopted during testing and the different laboratory techniques.

Limitations of the present study that it is an in-vitro study, where clinical variables cannot be accurately reproduced. The silicon replica technique provides 2D measurements. Further in-vivo studies are encouraged to evaluate the clinical results of different fabrication techniques under clinical ageing conditions on the internal adaptation of ceramometallic restorations using 3D evaluation technique.

## Conclusion

Within the study’s limitation, it can be concluded that different construction techniques of ceramometallic restoration were considered reliable regarding their internal fit and microleakage.

### Financial sponsorship

All materials and methods were funded by main researcher. Publication fees by UBT University.
